# Reconstruction of a massive abdominal wall defect after colon cancer surgery using bilateral thoracoabdominal flaps and a vertical rectus abdominis myocutaneous flap: a case report

**DOI:** 10.3389/fonc.2025.1698546

**Published:** 2025-11-17

**Authors:** Shuo Li, Zhi Zhang, Chongqing Gao, Tao Wang, Zhen Zhang, Zikang Wang, Guixiang Zhang, Gangcheng Wang

**Affiliations:** Department of Abdominal and Pelvic Tumor Surgery, The First Affiliated Hospital of Zhengzhou University, Zhengzhou, China

**Keywords:** colon cancer, tumor recurrence, abdominal wall reconstruction, vertical rectus abdominis myocutaneous flap, V-Y advancement flap, mesh

## Abstract

A 35-year-old woman presented with a local recurrence at the stoma site three months after Hartmann’s procedure for sigmoid colon cancer. In the subsequent months, the tumor exhibited rapid progression, with direct invasion into the abdominal wall. Her clinical course was characterized by persistent pain, fever, foul odor, and defecation disorder. The substantial disease burden ​​confined her to bed, leaving her unable to care for her two young children​​. Following a multidisciplinary team (MDT) evaluation, she was deemed a candidate for reoperative surgery. The procedure comprised en bloc resection of the recurrent tumor, followed by restoration of bowel continuity with a primary colorectal anastomosis and immediate abdominal wall reconstruction. The patient’s postoperative recovery was uneventful, with a marked improvement in quality of life. This case highlights the critical role of flap-based reconstruction in managing complex abdominal wall defects.

## 1 Introduction

Local recurrence after colorectal cancer surgery is associated with​​ factors such as tumor stage and surgical margin status (1). Local recurrence can develop at various sites, including the anastomosis, lateral pelvic wall, or stoma (2). The reconstruction of complex abdominal wall defects following extensive resection of locally recurrent tumors poses a major surgical challenge, and flap transplantation represents a pivotal reconstructive technique for such cases (3). This article reports a case of peristomal recurrence following surgery for sigmoid colon cancer. The extensive abdominal wall defect resulting from the extended resection was reconstructed with a combination of bilateral thoracoabdominal wall flaps and a pedicled vertical rectus abdominis myocutaneous (VRAM) flap.

## 2 Case presentation

On February 16, 2024, the patient presented to a local hospital with lower abdominal dull pain, diarrhea, and fever with no apparent cause. Emergency examination led to a diagnosis of sigmoid colon tumor perforation complicated by intestinal obstruction, and concurrent intestinal wall edema (involving the sigmoid colon and the intestinal wall adjacent to the perforation site) secondary to local inflammation and venous stasis. An emergency laparoscopic Hartmann’s procedure was performed. Postoperative pathological examination revealed a moderately to well-differentiated adenocarcinoma of the sigmoid colon, staged as pT3N1M0. The patient completed two cycles of the XELOX chemotherapy regimen in March and April 2024, respectively. A PET-CT scan in May 2024 indicated tumor recurrence at the stoma site. The chemotherapy regimen was subsequently adjusted multiple times. The patient was transferred to our center for further management eight months ago (February 2025).

**Figure 1:**
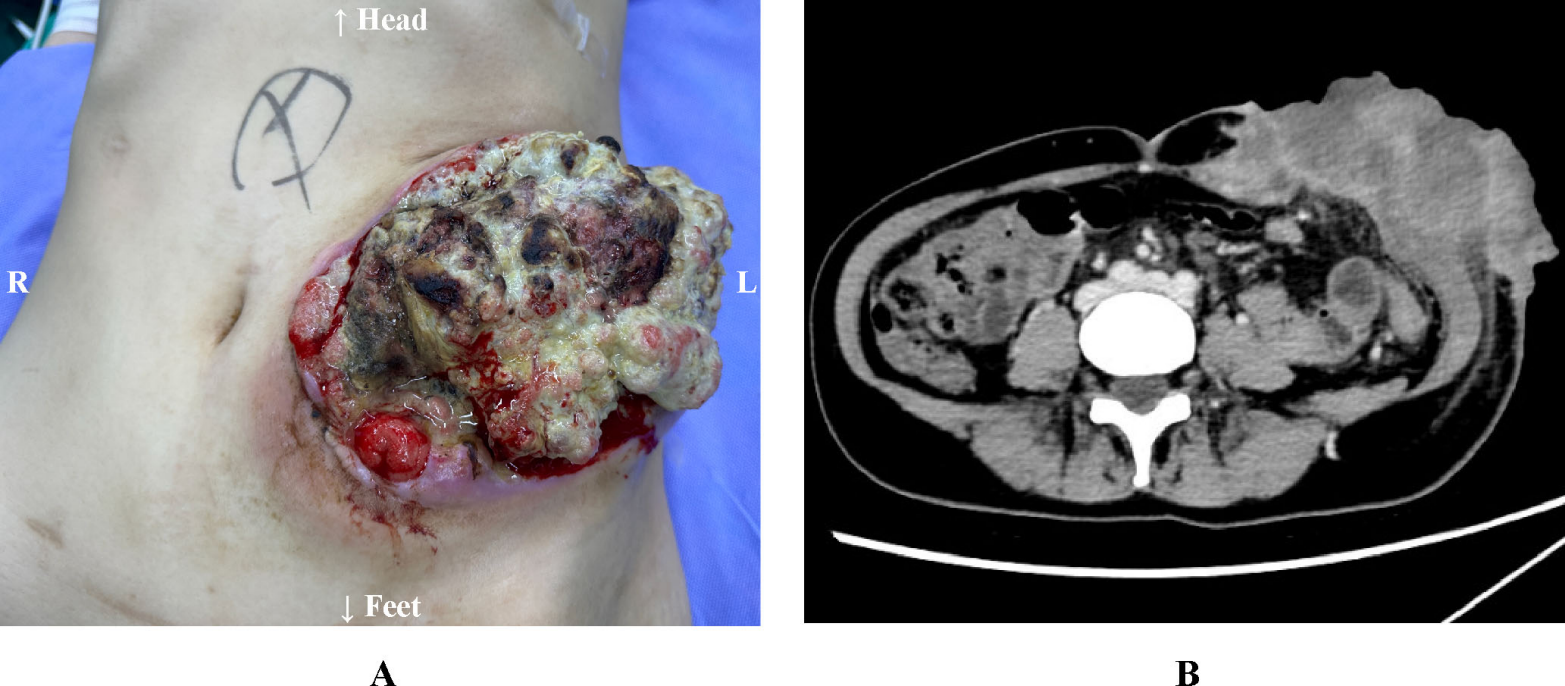
**(A)** The large tumor (approximately 10×10 cm) is associated with necrosis and hemorrhage. **(B)** Abdominal CT Scan findings (Preoperative, February 2025).

The patient had a history of two previous cesarean sections in 2014 and 2022. Family history was negative for hereditary colorectal cancer predisposition syndromes.​ Physical examination upon admission revealed a 15-cm transverse surgical scar on the abdomen. A tumor measuring approximately 10×10 cm was observed in the left lower quadrant, adjacent to the intestinal stoma. Since May 2024, ​​serial surveillance CT scans​​ have consistently demonstrated ​​progressive enlargement​​ of the recurrent tumor at the stoma site (**Figure 1**). The tumor was accompanied by foul odor, bleeding, and defecation disorder of the stoma. Laboratory tests revealed severe anemia (hemoglobin 55 g/L; reference range, 115–150 g/L), elevated carcinoembryonic antigen (CEA) at 22.50 ng/mL (reference range, 0–5 ng/mL), and elevated carbohydrate antigen 19-9 (CA19-9) at 88.50 U/mL (reference range, 0–37 U/mL). Cardiac and pulmonary functions, along with coagulation parameters, were within normal limits.

**Figure 2:**
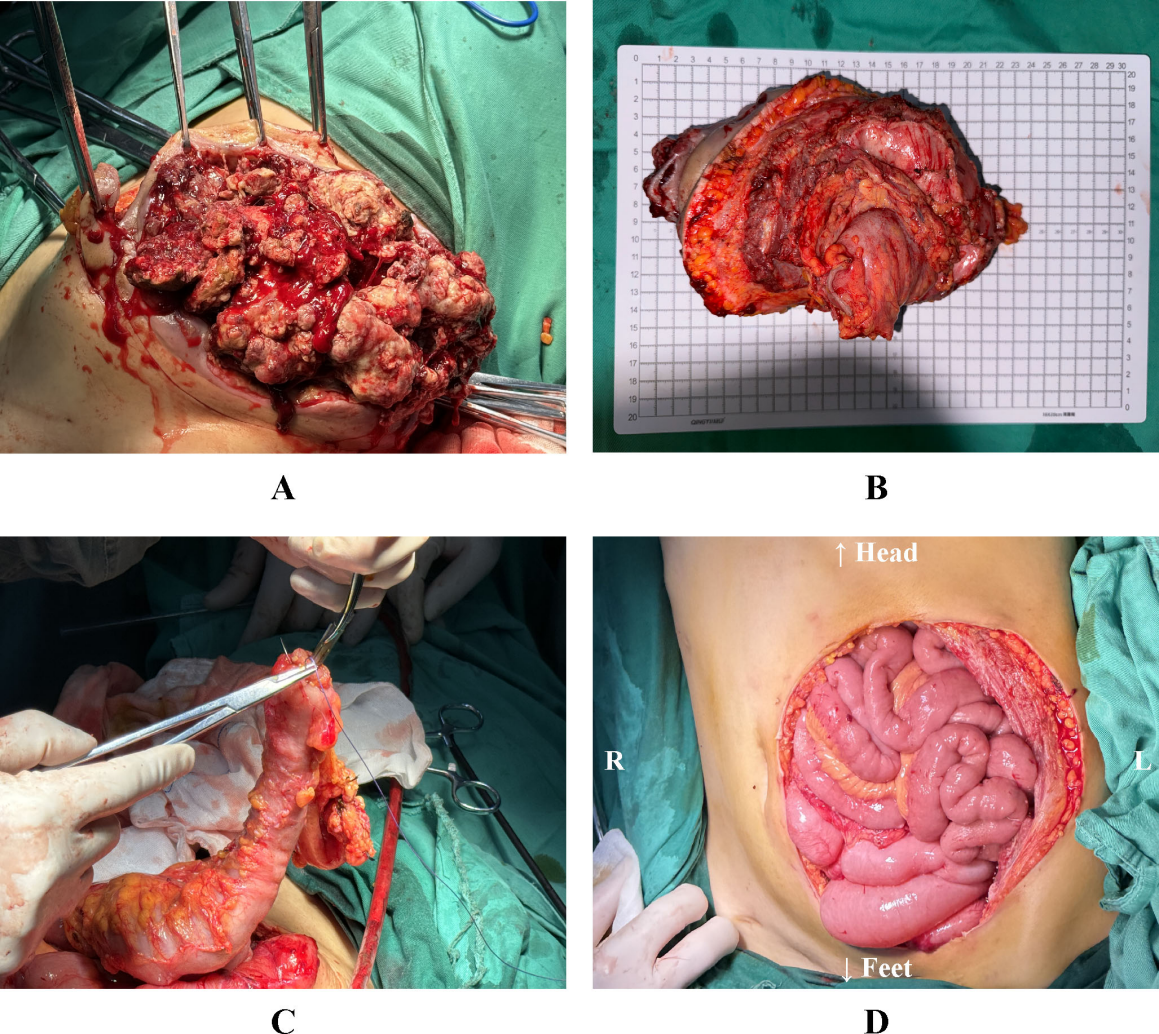
**(A)** Resection of the tumor and the affected abdominal wall. **(B)** Resected abdominal wall tumor specimen, including invaded abdominal wall tissue and bowel. **(C)** The freed distal end of the descending colon is being introduced into the anvil of a circular stapler. **(D)** The reconstructed alimentary tract and the left lower abdominal wall defect. L: The patient’s left side; R: The patient’s right side.

Following MDT discussions that deemed surgical intervention the optimal approach, the procedure was performed under general anesthesia one week later. Intraoperative exploration identified a large local recurrence at the stoma site, with invasion into the rectus abdominis muscle and the underlying bowel, accompanied by abdominal adhesions and ascites. First, an en bloc resection of the recurrent tumor and the involved abdominal wall was performed. The sigmoid colon was transected with an adequate margin from the tumor using a linear stapler. Finally, the intra-abdominal tumor component was completely resected en bloc, which included excision of the invaded left-sided partial rectus abdominis muscle and sigmoid colon. This resulted in an abdominal wall defect of approximately 180 cm². To achieve a tension-free anastomosis during digestive tract reconstruction, the splenic flexure and descending colon were fully mobilized. An end-to-side anastomosis was then performed between the distal end of the descending colon and the lateral wall of the upper rectal stump (**Figure 2**).

To ensure complete resection, the extent of invasion was initially assessed through intraoperative palpation. Pathological evaluation via frozen section analysis of the surgical margins confirmed no residual tumor.

**Figure 3:**
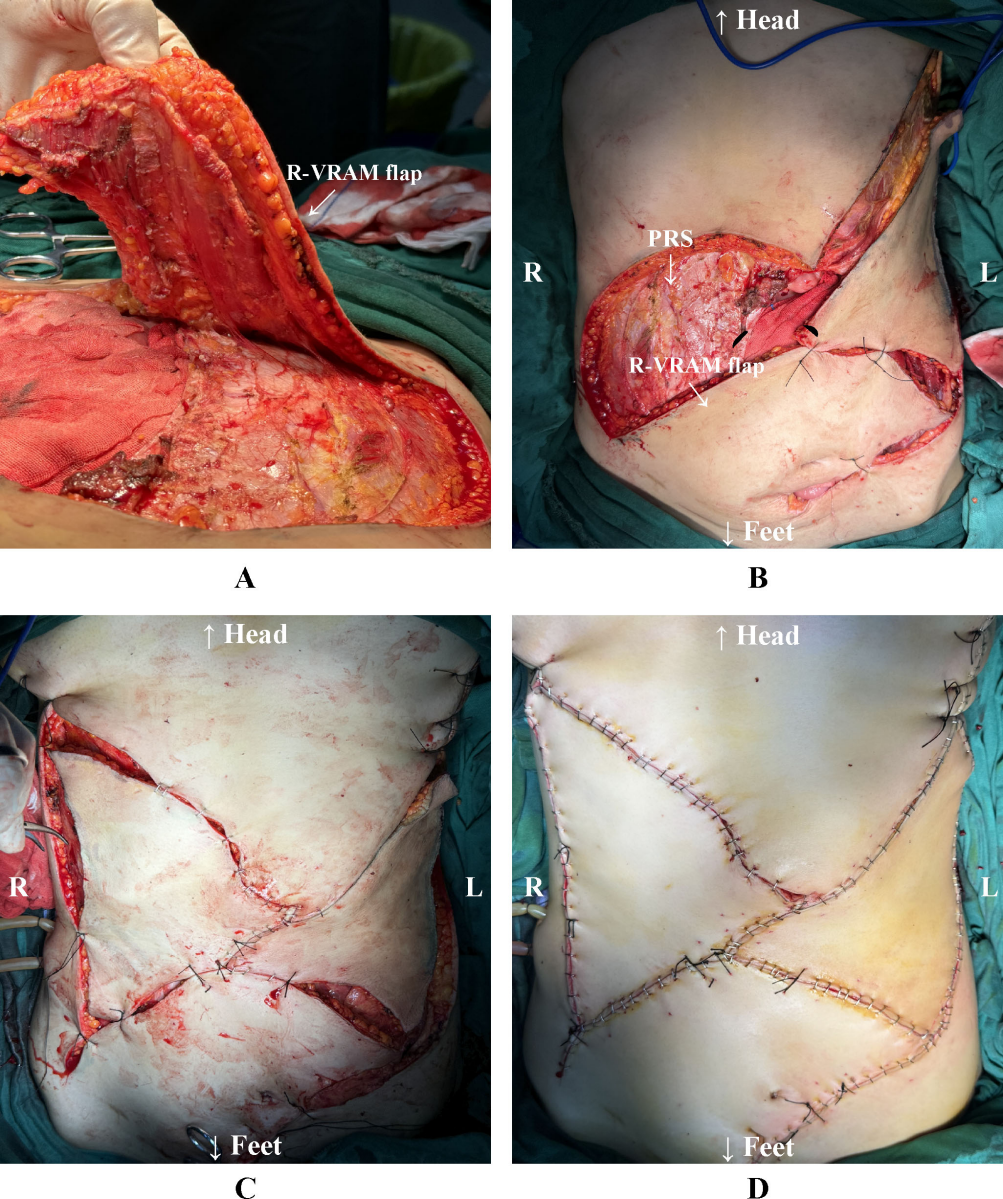
**(A)** Preparation of the right-sided pedicled VRAM flap. **(B)** The VRAM flap is rotated to the left toward the lower edge of the abdominal wall defect. The right-sided donor site preserves the posterior rectus sheath. **(C)** The VRAM flap was preliminarily sutured to the lower edge of the defect. The bilateral V-Y advancement flaps were preliminarily sutured. **(D)** All flaps were completely sutured and reinforced with staples. L: The patient’s left side. R: The patient’s right side. R-VRAM flap: Right-sided vertical rectus abdominis myocutaneous flap; PRS: Posterior rectus sheath.

Subsequently, the right-sided pedicled VRAM flap based on the inferior epigastric artery was ​​raised​​. The superior border of the VRAM flap extended to the right costal margin, the inferior border reached the superior border of the pubis, the medial border was at the abdominal wall defect, and the lateral border extended to the anterior axillary line. The VRAM flap was ​​elevated​​ in its entirety ​​with preservation of​​ the posterior sheath and peritoneum at the donor site. It was then ​​rotated to the left​​ and ​​advanced​​ to cover the defect in the left lower abdomen. After ​​provisional fixation​​ of the flap, ​​adequate coverage​​ was ​​confirmed​​. ​​According to the actual defect size​​, bilateral V-Y advancement flaps were designed along the inferior margins of the bilateral costal arches. These flaps were then ​​mobilized​​ to repair the residual defect by exploiting their ​​inherent elasticity and mobility​​. Throughout the procedure, meticulous ​​attention was paid to preserving​​ the blood supply to both flaps. The bilateral V-Y advancement flaps were ​​sutured in layers​​ to the inverted pedicled VRAM flap and the surrounding abdominal wall. Skin approximation was ​​reinforced​​ with surgical staples (**Figure 3**).

Histopathological examination confirmed a moderately differentiated adenocarcinoma. The tumor invaded through the intestinal wall into the adjacent skeletal muscle and subcutaneous adipose tissue. Lymph node metastasis was identified in one of eight lymph nodes examined (1/8). Postoperative laboratory tests at 10 days revealed a decline in tumor markers: CEA decreased to 2.90 ng/mL (within normal limits, 0–5 ng/mL), and CA 19–9 decreased to 46.80 U/mL. Adjuvant therapy was recommended upon complete healing of the abdominal wound.

**Figure 4:**
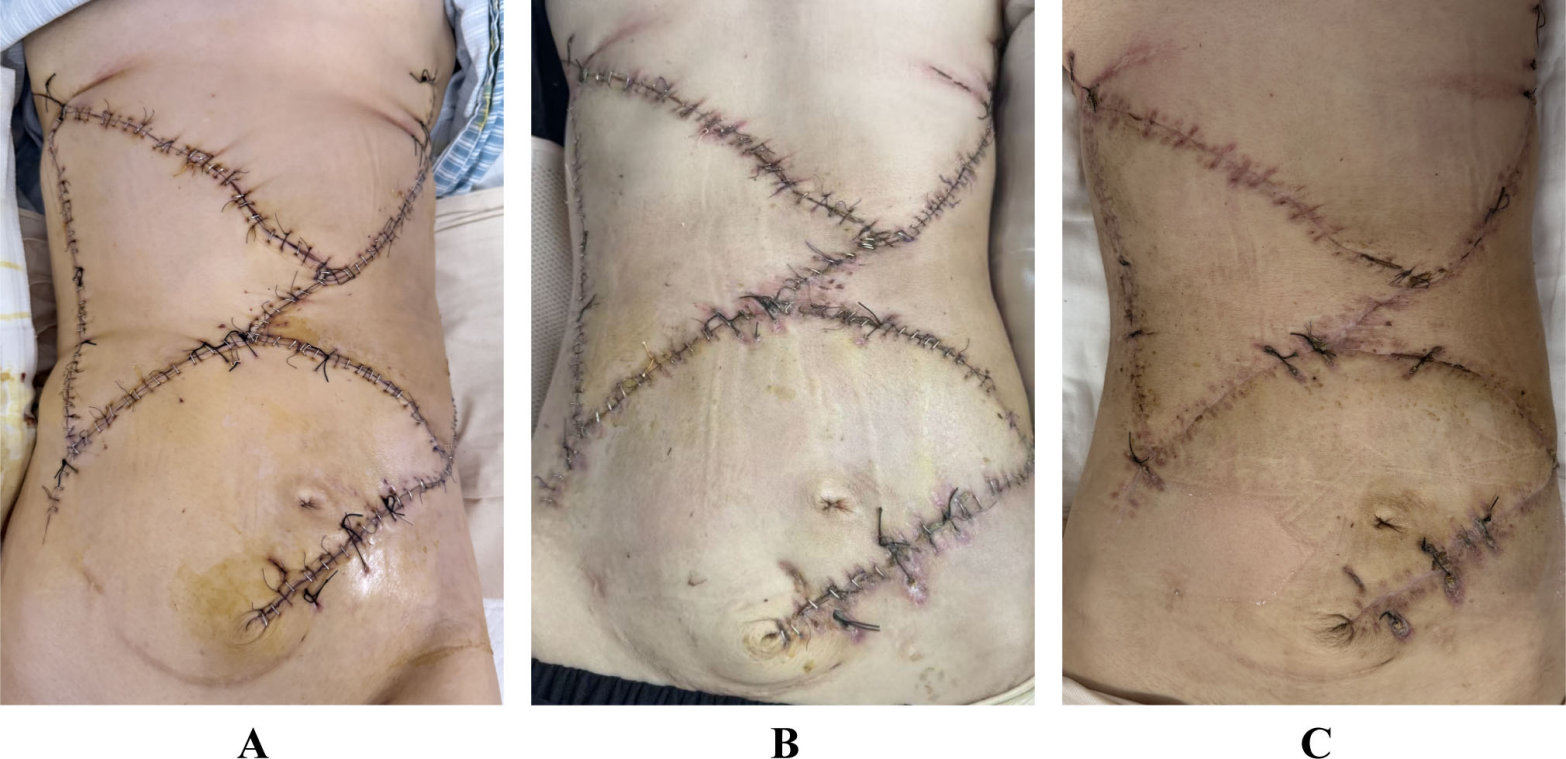
**(A)** Postoperative appearance of the flaps at 1 week. **(B)** Postoperative appearance of the flaps at 1 month. **(C)** Postoperative appearance of the flaps at 3 months.

The patient presented with marked malnutritional status and severe anemia attributable to chronic tumor-related catabolism. A comprehensive postoperative management protocol was implemented, involving personalized nutritional support and systematic wound care. The patient was successfully discharged one month following the procedure. During the 8-month follow-up period, serial contrast-enhanced CT scans confirmed no evidence of tumor recurrence or incisional hernia, and the surgical flaps remained fully viable without any signs of infection​​ (**Figure 4**).

## 3 Discussion and conclusions

This article reports a case in which a composite autologous tissue reconstruction strategy utilizing a pedicled VRAM flap combined with bilateral V-Y advancement flaps was employed.

Several well-established flap techniques are available for repair, each selected based on the defect’s size, location, and composition. The ​​Anterolateral Thigh (ALT) flap​​ can be harvested as a pedicled or free flap. The pedicled version, based on the descending branch of the lateral circumflex femoral artery, is ideal for reconstructing ipsilateral inguinal, perineal, and lower abdominal defects within a rotational arc. The free flap version allows for distant reconstruction through microvascular anastomosis (4, 5). The VRAM flap​​ is particularly valuable for reconstructing large, complex defects of the abdominal wall, pelvis, and perineum, owing to its reliable vascular pedicle and substantial tissue bulk. It is often reinforced with a mesh to provide additional structural support and reduce hernia recurrence (6, 7). In contrast, the ​​Deep Inferior Epigastric Artery Perforator (DIEP) flap​​ preserves the rectus abdominis muscle, making it suitable for soft tissue coverage but less ideal for defects requiring muscle volume or dynamic support (8). The ​​Superficial Circumflex Iliac Artery Perforator (SCIP) flap​​ is well-suited for skin and soft tissue defects in the inguinal region, lower abdomen, and perineum. It can be transferred as a pedicled or free flap (9).

We ultimately opted for a pedicled VRAM flap to repair the abdominal wall defect. This approach provides a robust vascular supply and substantial tissue volume, both critical for repairing large defects in a non-sterile environment. Additionally, the absence of microvascular anastomosis shortens the surgical duration. The ALT flap was considered suboptimal for several anatomic reasons. Furthermore, the ALT flap often possesses a variable and substantial subcutaneous adipose layer, which can lead to excessive bulk and contour irregularity at the recipient site (10). The SCIP flap, while useful for smaller defects, provides limited tissue volume and was therefore inadequate for a defect of this magnitude.

Mesh is a standard intervention in abdominal wall reconstruction for providing mechanical support (11). We acknowledge the significant value of using mesh. The decision to avoid mesh reinforcement was based on the patient’s individual circumstances, including ​​financial constraints​​ and the potential need for future reoperation in case of tumor recurrence. The presence of a foreign body could complicate subsequent surgery by promoting significant scar tissue formation (12). Moreover, absorbable meshes may lose mechanical strength over time, while non-absorbable meshes can provoke chronic foreign body reactions and pose risks of adhesion. Therefore, an autologous tissue-based reconstruction was ultimately performed. We also recommend that patients wear the abdominal binder long-term.

Following rotation and initial fixation of the pedicled VRAM flap, assessment indicated that complete coverage of the abdominal wall defect had not been achieved. To address the residual defect, bilateral V-Y advancement flaps were designed along the inferior margins of the thoracic wall. The V-Y advancement flap technique is particularly valuable in this context as it harnesses the inherent elasticity and extensibility of the skin and subcutaneous tissue (13). This approach enables primary closure of the donor site defect, making it particularly suitable for repairing moderate to large soft tissue defects (14). The combined use of the pedicled VRAM flap and bilateral V-Y advancement flaps ultimately achieved stable reconstruction of the extensive abdominal wall defect.

The patient was young with a relatively localized tumor, making complete resection theoretically feasible. Her family role also provided significant motivation for treatment. Faced with a large abdominal wall defect and the need to avoid using a mesh, we opted for the pedicled VRAM combined with bilateral V-Y advancement flaps to reconstruct the abdominal wall. The patient recovered well postoperatively. We believe this method of flap transplantation to repair abdominal wall defects has certain clinical reference value.

This article presents a case in which a composite autologous tissue reconstruction was performed using a pedicled VRAM flap combined with bilateral V-Y advancement flaps. The focus is on outlining the clinical decision-making process and therapeutic outcomes associated with this technique.

## Data Availability

The raw data supporting the conclusions of this article will be made available by the authors, without undue reservation.
